# Multi-parameter MRI radiomic features may contribute to predict progression-free survival in patients with WHO grade II meningiomas

**DOI:** 10.3389/fonc.2024.1246730

**Published:** 2024-06-28

**Authors:** Qiang Zeng, Zhongyu Tian, Fei Dong, Feina Shi, Penglei Xu, Jianmin Zhang, Chenhan Ling, Zhige Guo

**Affiliations:** ^1^ Department of Neurosurgery, Second Affiliated Hospital of Zhejiang University School of Medicine, Hangzhou, Zhejiang, China; ^2^ Department of Neurosurgery, Clinical Research Center for Neurological Diseases of Zhejiang Province, Hangzhou, China; ^3^ Department of Radiology, Second Affiliated Hospital of Zhejiang University School of Medicine, Hangzhou, Zhejiang, China; ^4^ Department of Neurology, Sir Runrun Shaw Hospital of Zhejiang University School of Medicine, Hangzhou, Zhejiang, China; ^5^ Department of Neurosurgery, Guangdong Provincial People’s Hospital (Guangdong Academy of Medical Sciences), Southern Medical University, Guangzhou, Guangdong, China

**Keywords:** meningioma, radiomics, progression-free survival, machine learning, MRI

## Abstract

**Aim:**

This study aims to investigate the potential value of radiomic features from multi-parameter MRI in predicting progression-free survival (PFS) of patients with WHO grade II meningiomas.

**Methods:**

Kaplan–Meier survival curves were used for survival analysis of clinical features. A total of 851 radiomic features were extracted based on tumor region segmentation from each sequence, and Max-Relevance and Min-Redundancy (mRMR) algorithm was applied to filter and select radiomic features. Bagged AdaBoost, Stochastic Gradient Boosting, Random Forest, and Neural Network models were built based on selected features. Discriminative abilities of models were evaluated using receiver operating characteristics (ROC) and area under the curve (AUC).

**Results:**

Our study enrolled 164 patients with WHO grade II meningiomas. Female gender (*p*=0.023), gross total resection (GTR) (*p*<0.001), age <68 years old (*p*=0.023), and edema index <2.3 (*p*=0.006) are protective factors for PFS in these patients. Both the Bagged AdaBoost model and the Neural Network model achieved the best performance on test set with an AUC of 0.927 (95% CI, Bagged AdaBoost: 0.834–1.000; Neural Network: 0.836–1.000).

**Conclusion:**

The Bagged AdaBoost model and the Neural Network model based on radiomic features demonstrated decent predictive ability for PFS in patients with WHO grade II meningiomas who underwent operation using preoperative multi-parameter MR images, thus bringing benefit for patient prognosis prediction in clinical practice. Our study emphasizes the importance of utilizing advanced imaging techniques such as radiomics to improve personalized treatment strategies for meningiomas by providing more accurate prognostic information that can guide clinicians toward better decision-making processes when treating their patients’ conditions effectively while minimizing risks associated with unnecessary interventions or treatments that may not be beneficial.

## 1 Introduction

Meningiomas constitute 38.3% of all tumors of the central nervous system, and the annual incidence rate is approximately 8 per 100,000 individuals, rendering them the most prevalent primary intracranial neoplasms (1). According to the grading criteria adopted in the 2021 World Health Organization (WHO) guidelines, meningiomas can be classified into grades I through III (2). The majority of meningiomas are benign (WHO grade I), while 20% of meningiomas are high-grade (WHO grades II and III), which exhibit a greater propensity for recurrence and worse prognosis (3, 4). In the previous 2016 WHO guidelines, the pathological diagnostic criteria for WHO grade II meningioma were substantially revised; specifically, brain invasion was adopted as a novel standard for the diagnosis of WHO grade II meningioma (2). This has led to the increased diagnosis of WHO grade II meningioma in some patients previously diagnosed with WHO grade I meningioma, resulting in a significant change in the incidence rate of WHO grade II meningioma (5). Therefore, previous studies on the prognosis of patients with WHO grade II meningioma may no longer be applicable, and further research is warranted.

In clinical practice, surgery constitutes the primary treatment for symptomatic WHO grade II meningiomas or those expected to become symptomatic in the near future. The goal of the operation is to achieve gross total resection (GTR), specifically Simpson grades I through III resection, which can cure most patients with meningioma (6). However, GTR is not always feasible and may be limited by various factors, including tumor location, venous sinus, and neurovascular tissue involvement. These factors may affect the surgical technique and the extent of resection, which are closely related to tumor recurrence and progression (7). Even after undergoing GTR, WHO grade II meningiomas still exhibit a high rate of progression, with a 30%–40% rate of progression within 5 years (6). Previous studies have reported several factors associated with postoperative progression of WHO grade II meningiomas, including extent of resection, age, postoperative radiotherapy, and tumor location (8–10). Molecular biomarkers such as PTTG1, LEPR, and TERT have also been shown to correlate with shortened progression-free survival (PFS) (11, 12). However, predicting the progression of WHO grade II meningioma is not always effective based solely on clinical features. Moreover, molecular biomarkers have not been widely implemented. The existing prognostic methods and models mainly rely on clinical information and features and incorporate qualitative or semi-quantitative semantic features in medical imaging. In this study, we used radiomics as a novel approach, which is to extract high-dimensional radiomics features, conduct quantitative analysis, and construct more accurate predictive models.

Radiomics constitutes an emerging research field that aims to calculate and extract multidimensional features containing useful information from radiographic medical images (X-ray, CT, and MRI) and perform various quantitative analyses (13–15). Previous radiomic studies of meningiomas primarily focused on preoperative grading (16–22). Some research investigated prognosis and relapse of meningioma following surgery (23–25). In this study, we aim to develop predictive models using radiomic features and artificial intelligence for predicting PFS in patients with WHO grade II meningiomas.

## 2 Methods

Owing to the retrospective nature of this research and the anonymous, non-identifiable imaging data, the Institutional Review Board at our hospital approved this study and waived the requirement for written informed consent.

### 2.1 Patients

Patients who underwent surgery and received a histopathological diagnosis of WHO grade II meningioma between January 2013 and September 2020 were retrospectively reviewed and enrolled in our cohort. Corresponding preoperative images including T1-weighted imaging (T1WI), T2-weighted imaging (T2WI), and T1 contrast-enhanced (T1CE) sequences were exported and stored using the Picture Archiving and Communication System at our hospital. The following patients were excluded to minimize heterogeneity within the cohort (1): loss of follow-up or failure to provide relevant information (2); follow-up time <5 years with no observed progression (3); incomplete preoperative images (absence of either axial T1WI and T2WI of T1CE sequence) (4); presence of significant image artifacts (5); prior radiotherapy or surgery (6); multiple intracranial meningiomas (7); non-newly diagnosed meningioma; and (8) spinal meningiomas. Meningiomas were reclassified according to the 2021 WHO classification system (2).

### 2.2 Image acquisition

Preoperative brain MRI images were all captured in our hospital. MRI scanners are as follows: 3.0-T uMR 780 scanner (United Imaging, Shanghai, China), 3.0-T Discovery MR750w, 3.0-T Discovery MR750, 1.5-T Signa HDxt and 1.5-T Signa Excite scanners (GE Healthcare, Chicago, IL, USA), 1.5-T MAGNETOM Avanto, 1.5-T MAGNETOM Aera, and 1.5-T Sonato scanners (Siemens Healthineers, Erlangen, Germany). Through a peripheral venous catheter, pre-contrast Gadodiamide (Omniscan; GE Healthcare, Chicago, IL, USA) was administered at a dose that was standardized depending on body weight (0.2 ml/kg body weight, up to a maximum of 20 ml).

### 2.3 Clinical information collection and follow-ups

Corresponding demographic and clinical data, encompassing age, gender, tumor location, degree of peritumoral edema, and extent of resection, were archived and retrieved via the Hospital Information System (HIS) at our institution.

Criteria for tumor location are as follows (1): skull base meningioma, the neoplasm contacts or invades the anterior skull base or clivus in any imaging slice preoperatively (2); parasagittal meningioma, the tumor contacts or invades the sagittal sinus in any preoperative imaging slice (3); convexity meningiomas, lesions located on the convexity of the brain excluding proximity to the skull base, venous sinus, cerebral ventricles, or cerebellum; and (4) meningiomas at other locations, meningiomas apart from those aforementioned.

The magnitude of peritumoral edema was characterized utilizing the Edema Index (EI) as an evaluative benchmark (26). The EI was calculated as the sum of edema volume and tumor volume divided by tumor volume. Severe edema was defined as peritumoral edema volume >50% of tumor volume; otherwise, it was defined as non-severe.

The Simpson grade was applied to determine the extent of surgical resection, evaluated based on information from the HIS. Simpson grades I–III were considered GTR, while Simpson grades IV–V were deemed subtotal resection (STR) (27).

Data collected during follow-up included the following (1): patient survival status (2); date of most recent re-examination (3); whether meningioma recurrence transpired; and (4) whether treatment was undertaken and the particular therapy following recurrence. Follow-up time was the interval between follow-up date and operation date, calculated in months. PFS time was the interval between operation date and date of recurrence or patient demise, calculated in months. In the absence of recurrence or death, follow-up time was considered PFS. If the patient did not undergo postoperative re-examination and did not perish, follow-up time was regarded as PFS. Enrolled patients were categorized into two groups: (a) progression group, patients with PFS ≤ 60 months who exhibited progression (including recurrence or death), and (b) non-progression group, patients with PFS > 60 months.

### 2.4 Image preprocessing

Image preprocessing entailed the application of a series of transformations to initial images to improve image quality and enable more reproducible and comparable statistical analysis. Image preprocessing was performed as delineated in our previous study (28). To facilitate further investigation, the SPM12 module of MATLAB R2020b (MathWorks, Natick, MA, USA) was utilized to convert original Digital Imaging and Communications in Medicine (DICOM) format images to Neuroimaging Informatics Technology Initiative (NIfTI) format. Several intrinsic factors that lead to inhomogeneity of magnetic resonance images may introduce error into the study, such as low frequency intensity non-uniformity and high frequency intensity variations. The N4ITK algorithm and Smallest Univalue Segment Assimilating Nucleus (SUSAN) algorithm were intended to compensate for the aforementioned variations, respectively (29, 30). The N4ITK and SUSAN algorithms were both embedded in the Cancer Imaging Phenomics Toolkit (CaPTk) software (version 1.8.1, Center for Biomedical Image Computing and Analytics, University of Pennsylvania, Philadelphia, Pennsylvania, USA) and were employed to preprocess image data (31, 32).

### 2.5 Segmentation and radiomic feature extraction

**Figure 1:**
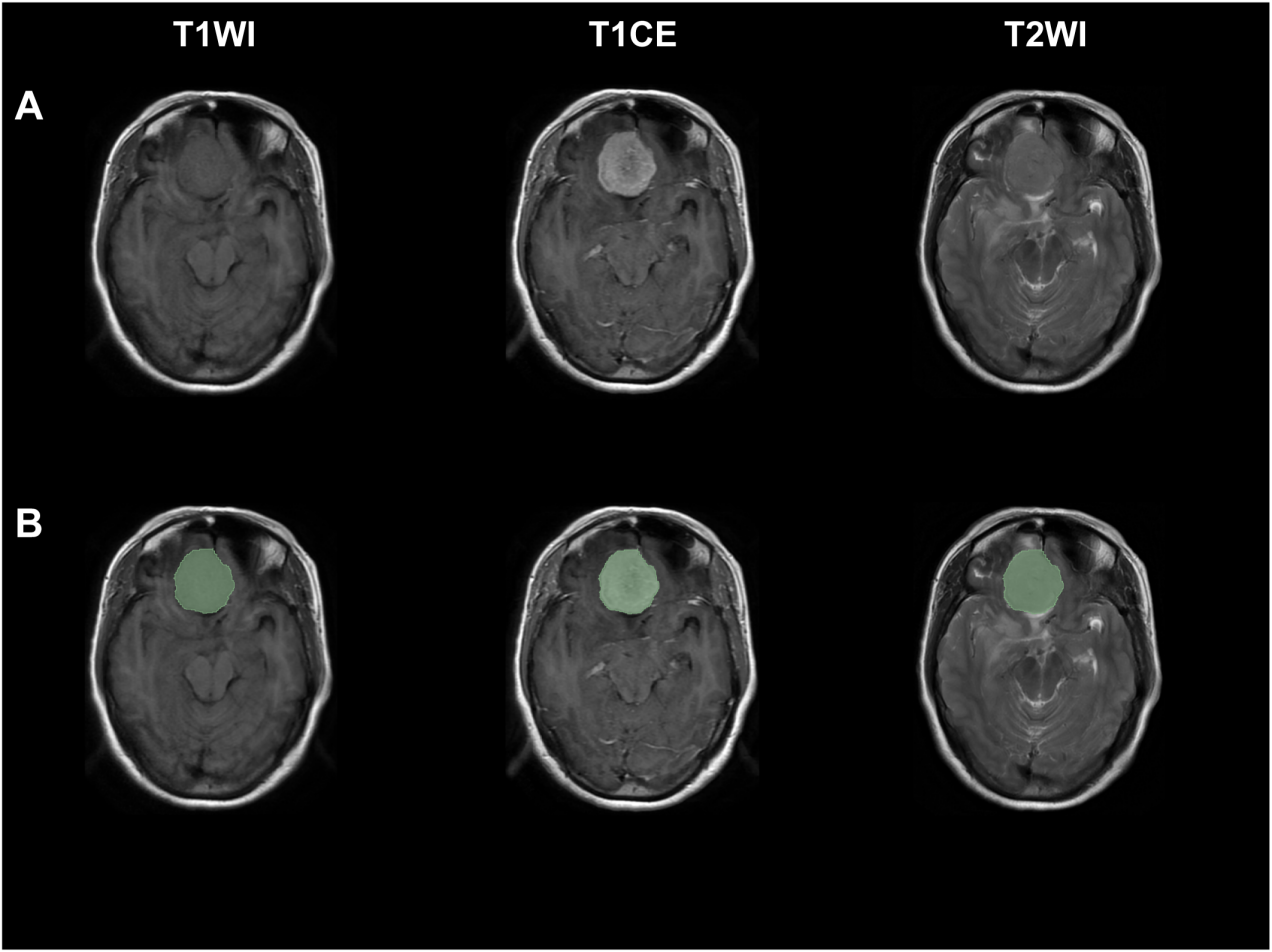
Exemplary figures demonstrated original images and corresponding ROI mask (green) of a patient with WHO grade II meningiomas from T1WI, T1CE, and T2WI sequences. **(A)** Original images, **(B)** original images with corresponding ROI mask.

Segmentation and feature extraction were performed utilizing 3D Slicer software (Surgical Planning Laboratory, Brigham and Women’s Hospital, Boston, MA, USA; http://www.slicer.org). Patients were reordered and renumbered; clinical data such as name, age, and grade were also blinded. To minimize the effect of different sampling between images and anisotropic sampling in different directions, image interpolation and resampling to 1 mm^3^ voxel resolution were first undertaken (33). The general registration (BRAINS) module embedded in 3D Slicer software was then employed to conduct within-subject registration of T1WI, T2WI, and T1CE utilizing linear transformation and rigid (6 degrees of freedom) mode. Segmentation was implemented on the tumor region of T1CE by two independent investigators, a neurosurgeon with 7 years of experience (QZ) and a radiologist with 10 years of experience (FD), using a semi-automated segmentation method. The threshold tool, intensity-based level tracing tool, and paint tool of 3D Slicer software were utilized during the segmentation process. Segmentation was censored by the investigators, with any disagreement resolved through discussion and consensus. Exemplary figures are displayed in **Figure 1**. Radiomic features were then calculated and extracted from T1WI, T2WI, and T1CE, respectively, using the corresponding produced volumes of interest and the PyRadiomics package (http://www.radiomics.io/pyradiomics.html). A total of 851 radiomic features were extracted for each patient from each sequence. Categories of radiomic features were (1) First Order Statistics (2), Shape-based (3), Gray Level Co-occurrence Matrix (4), Gray Level Size Zone Matrix (5), Gray Level Run Length Matrix (6), Neighboring Gray Tone Difference Matrix (7), Gray Level Dependence Matrix, and (8) Wavelet-based. Radiomic features from T1WI, T2WI, and T1CE were integrated for further investigation.

### 2.6 Feature processing and screening

To assess the stability of radiomics features, inter-observer reproducibility test was conducted. A total of 20 patients were randomly selected to redo the segmentation process as described above by another investigator with 3 years of experience (ZYT). This result was solely utilized to determine interobserver concordance by calculating intraclass correlation coefficient (ICC) values. ICC is a metric that quantifies the degree of consistency and–agreement in measurements. Research conducted by Perinetti et al. has demonstrated that an ICC value exceeding 0.9 is indicative of excellent consistency and stability in repeated measurements (34). Consistent with this finding, our research team has previously employed an ICC threshold of 0.9 for inter-observer reproducibility test in our published study (28). Consequently, features with an ICC ≥ 0.9 were deemed stable and thus chosen.

A succession of processing steps was carried out to curate raw data before further analysis. Z normalization was applied to reduce the influence of different feature magnitudes (35). Data skewness was addressed using Box–Cox transformation, and features with near-zero variance, which have few unique values and low frequencies, were also removed (36, 37). After data preprocessing, patients were randomly partitioned into training and test sets at a ratio of 8:2 according to meningioma progression. The Max-Relevance and Min-Redundancy (mRMR) algorithm was then implemented for feature selection and dimensionality reduction. The mRMR algorithm is an advanced approach based on the Max-Relevance algorithm proposed by Peng et al. in 2005 (38). The mRMR algorithm, grounded in the principles of mutual information theory, represents an embedded method for feature selection. Mutual information, a key concept in information theory, serves to quantify the degree of correlation between features, with higher values denoting stronger relationships. The mRMR algorithm combines both max-relevance and min-redundancy criteria, aiming to choose a feature that minimizes redundancy and maximizes relevance. By employing the mRMR algorithm, one can calculate the mutual information between each of the N features and the target variable, subsequently arranging them in a descending order based on these calculations. This ranked list is then utilized to select a subset of input features, ranging from 1 to N, as the preliminary input feature set. Feature selection was performed on the training set. R software (version 4.1.2; The R Foundation for Statistical Computing, Vienna, Austria; https://www.r-project.org/) was utilized to conduct the aforementioned analyses.

### 2.7 Statistical analysis

All statistical analyses were performed using R software. The training set was utilized to construct four supervised machine-learning techniques: Bagged AdaBoost, Stochastic Gradient Boosting, Random Forest, and Neural Network. The five-time repeated 10-fold cross-validation approach was employed, and model performance was assessed using the area under the curve (AUC) with 95% confidence intervals (CIs). Model performance was further validated on the test set. Receiver operating characteristic (ROC) curves were used to calculate Youden’s indexes, which were then exploited as threshold values. Confusion matrices and model performance metrics (accuracy, sensitivity, specificity, positive predictive value, and negative predictive value) were also computed using the corresponding threshold value. Feature importance for different models was estimated over 20 permutations, measured and represented by 1-AUC loss after permutation. Moreover, lift charts were generated as a visualization tool for evaluating the model’s ability to detect events in a dataset of binary classifications (39). To further explore the final models, analysis of ceteris paribus profiles and accumulated local profiles was also performed (40).

The difference of baseline characteristic between the training set and test set was examined using chi-squared tests and Mann–Whitney U-tests for categoric and continuous variables, respectively. Kaplan–Meier survival curves of clinical features were carried out for survival analysis. A *p*-value < 0.05 was considered statistically significant.

## 3 Results

### 3.1 Baseline characteristics

**Figure 2:**
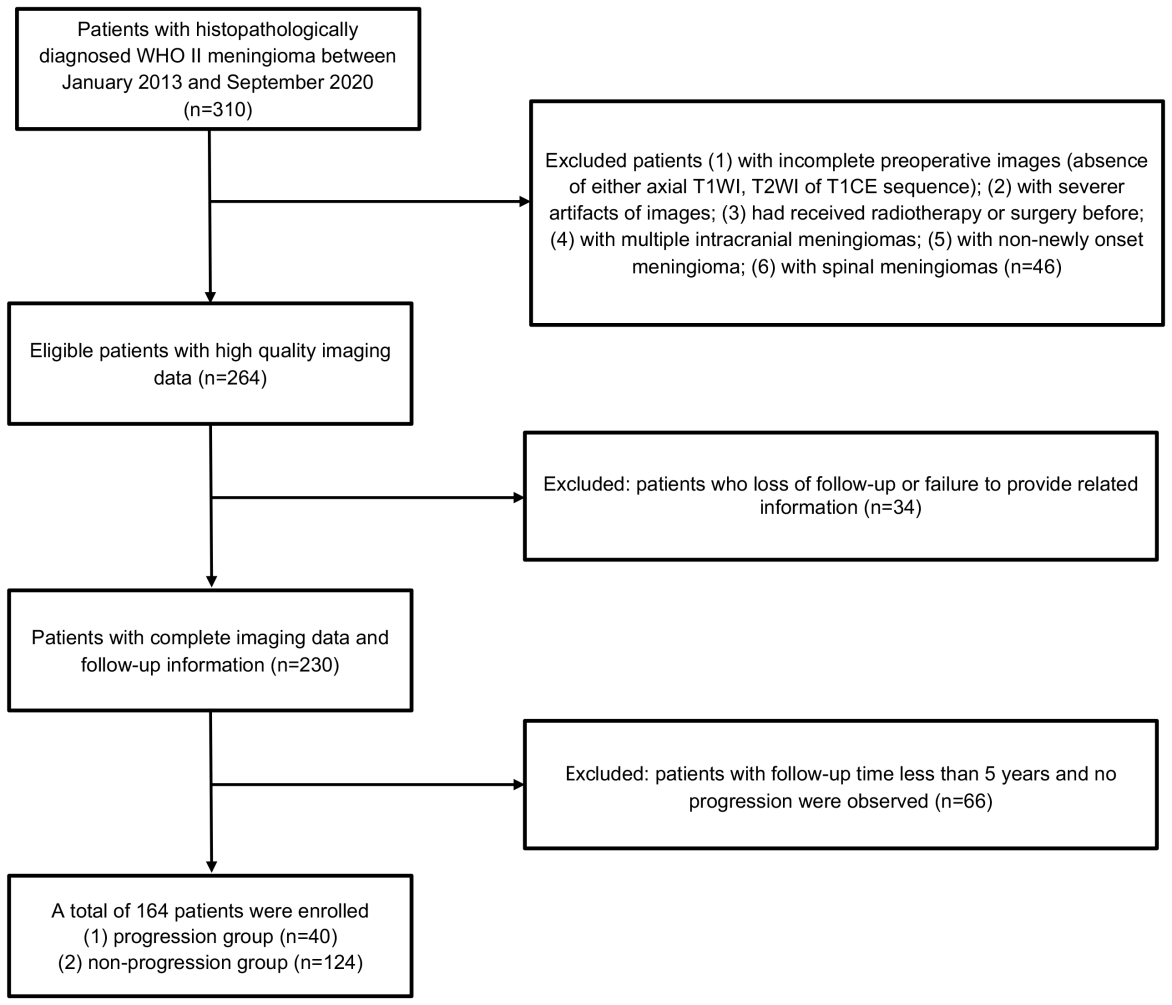
Flow chart of inclusion and exclusion processes for patients with WHO grade II meningiomas in our study.

Of the 310 patients, 164 were finally selected according to exclusion criteria and were enrolled in our cohort. The selection process and the number of patients excluded at each stage of the selection process are listed in **Figure 2**. Baseline clinical characteristics are shown in **Table 1**. Of all the patients, 93 (56.7%) patients were female, and 136 (82.9%) underwent GTR. The patients had a median [inter-quartile range (IQR)] age of 61.0 (54.8–69.0) years and showed a median (IQR) EI of 1.49 (1.03–2.61). A total of 132 and 32 patients were randomly allocated into training set and test set, respectively. Except for the extent of resection (*p* = 0.014 and 0.039 in training set and test set, respectively), there were no significant differences regarding age (*p* = 0.473 and 0.631 in training set and test set respectively), gender (*p* = 0.631 and 0.220 in training set and test set, respectively), tumor location (*p* = 0.128 and 0.300 in training set and test set, respectively), and EI (*p* = 0.159 and 0.260 in training set and test set, respectively) within each group (all *p* > 0.05).

**Table 1:** Baseline clinical characteristic of patients enrolled in this study.

Variables	Training set(n=132)		p	Test set(n=32)		*p*
	Non-progression	Progression group		Non-progression	Progression group	
Patients	100	32		24	8	
Age, years	62.5 [55.0–71.0]	63.0 [57.0–70.2]	0.473	57.0 [54.0–61.2]	58.0 [50.8–69.0]	0.631
Gender, No. (%)			0.631			0.220
Male	40 (40.00%)	15 (46.87%)		10 (41.7%)	6 (75.0%)	
Female	60 (60.00%)	17 (53.13%)		14 (58.3%)	2 (25.0%)	
Tumor location, No. (%)			0.128			0.300
Convexity	28 (28.0%)	3 (9.38%)		5 (20.8%)	0 (0.00%)	
Parasagittal	23 (23.0%)	11 (34.4%)		5 (20.8%)	4 (50.0%)	
Skull base	40 (40.0%)	14 (43.8%)		7 (29.2%)	3 (37.5%)	
Other	9 (9.00%)	4 (12.5%)		7 (29.2%)	1 (12.5%)	
EI	1.46 [1.03–2.36]	2.03 [1.08–3.02]	0.159	1.24 [1.00–1.76]	1.53 [1.12–3.03]	0.260
Extent of resection, No. (%)			0.014			0.039
GTR	87 (87.00%)	21 (65.63%)		23 (95.8%)	5 (62.5%)	
STR	13 (13.00%)	11 (34.37%)		1 (4.17%)	3 (37.5%)	

EI, Edema Index; GTR, gross total resection; STR, subtotal resection.

### 3.2 Survival analysis

**Figure 3:**
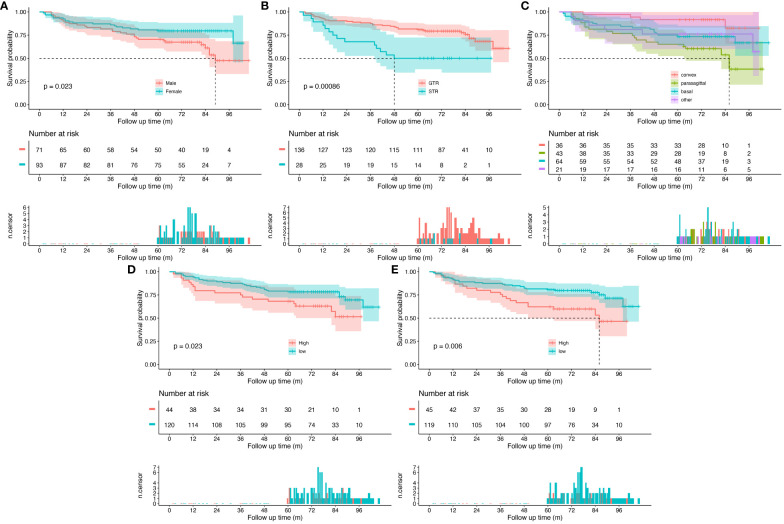
Kaplan–Meier survival curves for all patients. **(A)** Patients grouped by sex, **(B)** patients grouped by extent of resection, **(C)** patients grouped by tumor location, **(D)** patients grouped by age, and **(E)** patients grouped by Edema Index. The top panel shows survival curves with confidence interval for each group. The middle panel is risk table that shows the number of subjects at risk for each group at given follow time. The bottom panel is the number of censored subjects barplot, which shows the number of subjects is censored for each group at given follow time.

Survival analysis and Kaplan–Meier survival curves were performed for all patients based on clinical characteristic. Cutoff values were set to transfer continuous variables into categorical variables. Cutoff values for age and EI were 68 and 2.3, respectively. Kaplan–Meier survival curves are shown in **Figure 3**. Female (*p*=0.023), GTR (*p*<0.001), age<68 (*p*=0.023), and EI<2.3 (*p*=0.006) were demonstrated as protective factors for PFS of WHO grade II meningiomas. As for tumor location, pairwise comparisons log-rank test was carried out with *p*-values adjusted by the Bonferroni correction method, patients with convex meningiomas had a significant longer PFS than patients with parasagittal meningiomas (*p*=0.005), while no significant differences were observed in other pairs.

### 3.3 Feature selection and model construction

**Figure 4:**
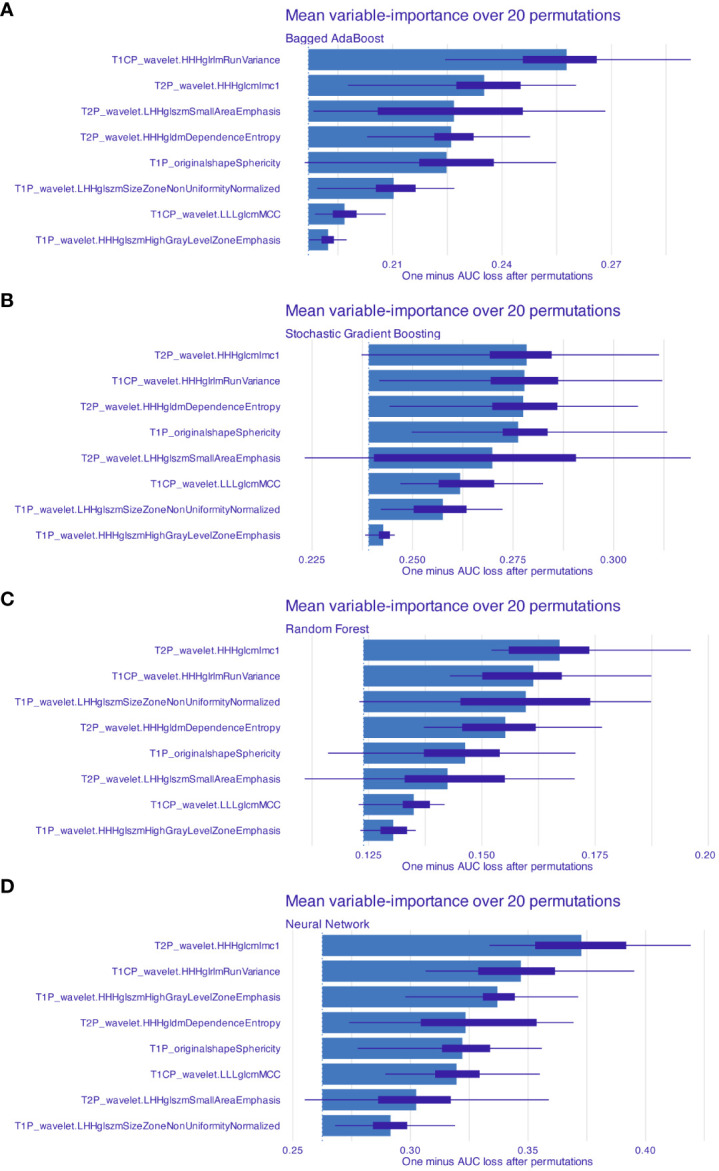
Features’ importance for models based on radiomics features. Mean feature importance for **(A)** Bagged AdaBoost, **(B)** Stochastic Gradient Boosting, **(C)** Random Forest, and **(D)** Neural Network was estimated over 20 permutations. Feature importance was measured and represented by 1-AUC loss after permutation. Features were arranged by descend order of importance.

For each patient, a total of 2,553 radiomic features from T1WI, T2WI, and T1CE underwent feature selection process; eight radiomic features were ultimately selected after ICC and mRMR algorithm (**Table 2**). Radiomic features from T1WI, T2WI, and T1CE were 3, 3, and 2, respectively. Seven of them were wavelet-based features; the remaining feature was shape based. Four supervised machine learning algorithms, including Bagged AdaBoost, Stochastic Gradient Boosting, Random Forest, and Neural Network, were exploited to construct prediction models based on different feature signatures. The importance of features for different models was calculated over 20 permutations, represented by 1-AUC (**Figure 4**). Among the four models, the HHHglcmlmc1 feature of Wavelet category from T2 sequence and HHHglrlmRunVariance feature of Wavelet category from T1C sequence remained the most two important features, while variations were observed in other radiomic features.

**Table 2:** Radiomic features selected by mRMR algorithm.

	Features
1	T2_wavelet.HHH_gldm_DependenceEntropy
2	T2_wavelet.HHH_glcm_Imc1
3	T1C_wavelet.HHH_glrlm_RunVariance
4	T2_wavelet.LHH_glszm_SmallAreaEmphasis
5	T1_original_shape_Sphericity
6	T1C_wavelet.LLL_glcm_MCC
7	T1_wavelet.HHH_glszm_HighGrayLevelZoneEmphasis
8	T1_wavelet.LHH_glszm_SizeZoneNonUniformityNormalized

### 3.4 Performance of models

**Figure 5:**
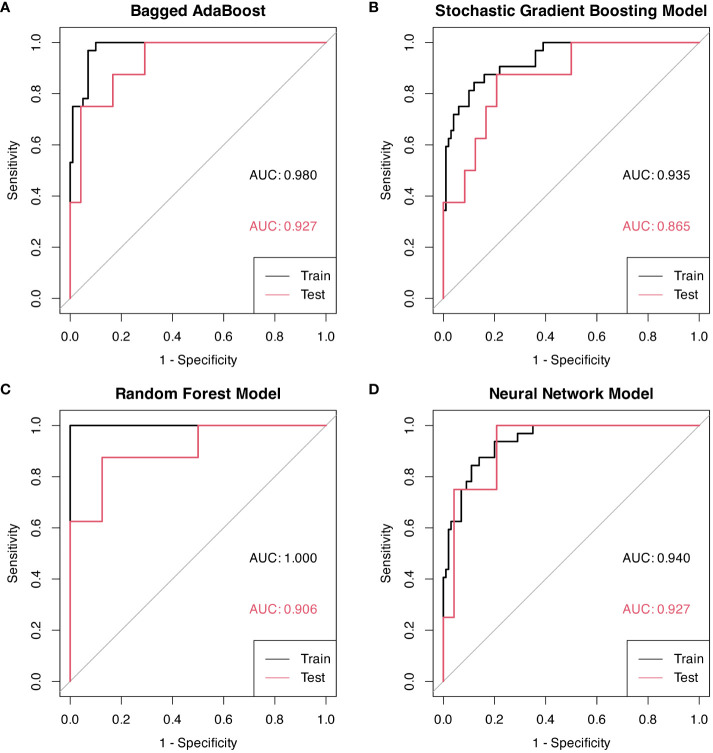
ROC curves for models based on radiomic features, each graph represents a different model. Youden indexes with associated sensitivity and specificity were displayed on ROC curves. **(A)** Bagged AdaBoost model on training and test sets. **(B)** Stochastic Gradient Boosting on training and test sets. **(C)** Random Forest model on training and test sets. **(D)** Neural Network model on training and test sets.

**Figure 6:**
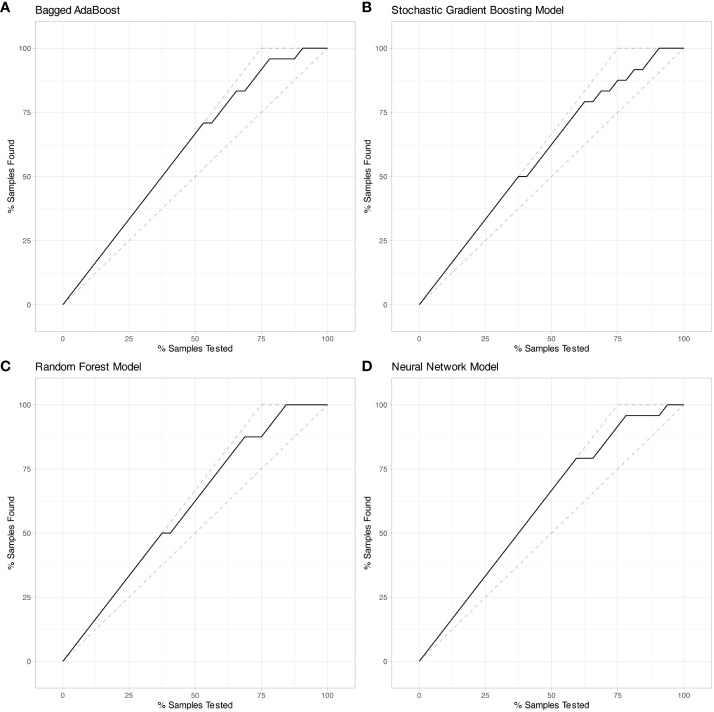
Lift charts on test set exhibited Bagged AdaBoost, Stochastic Gradient Boosting, Random Forest, and Neural Network models based on radiomic features. **(A)** Bagged AdaBoost model, **(B)** Stochastic Gradient Boosting model, **(C)** Random Forest model, and **(D)** Neural Network model. Each line displays the percentage of samples that were discovered out of the total samples analyzed.

Performance of models were represented by ROC curves and corresponding AUC values (**Figure 5**). All four machine learning models exhibited decent performance on test set with AUC>0.84. The Bagged AdaBoost model and the Neural Network model achieved the best performance on test set. The Bagged AdaBoost model has an AUC of 0.927 (95% CI, 0.834–1.000), an accuracy of 87.5% (95% CI, 0.710–0.965), a sensitivity of 100.0%, and a specificity of 83.3%. The Neural Network model has an AUC of 0.927 (95% CI, 0.836–1.000), an accuracy of 84.4% (95% CI, 0.672–0.947), a sensitivity of 100.0%, and a specificity of 79.2%. Besides AUC values, Bagged AdaBoost and Random Forest both achieved a peak accuracy of 87.5% on the test dataset. However, the imbalance in the distribution of progression versus non-progression patient samples curtails the explanatory validity of accuracy for the predictive models. Furthermore, the sensitivity and specificity profiles of the various predictive models present unique strengths; Bagged AdaBoost and Neural Network both exhibited perfect sensitivity. In the realm of specificity, Random Forest emerged as the leader among the four models, with a specificity of 87.5%. The differential emphasis on sensitivity and specificity across models also provides us with valuable practical guidance, informing our selection of the most appropriate model based on its relative strengths in sensitivity or specificity for predicting diverse target categories. Lift charts were also employed to demonstrate the discriminate ability of models to detect events in the dataset (**Figure 6**). Furthermore, several related model indicators were calculated, and elaborate performance metrics, including accuracy, kappa, sensitivity, specificity, positive predictive value, and negative predictive value of final models on the training set and test set were aggregated in **Table 3**. Confusion matrices were calculated using corresponding Youden index and were visualized by waterfall plots (**Supplementary Figure S1**).

**Table 3:** Performance metrics of prediction models on training set and test set.

Subset	Models	AUC (95% CI)	Accuracy (95%CI)	Kappa	Sensitivity	Specificity	PPV	NPV
Training	Bagged AdaBoost	0.980 (0.963–0.998)	92.4% (0.865–0.963)	81.36%	100.00%	90.00%	76.19%	100.00%
	Stochastic Gradient Boosting	0.935 (0.890–0.980)	87.1% (0.802–0.923)	67.36%	84.38%	88.00%	69.23%	94.62%
	Random Forest	1.000 (1.000–1.000)	100.0% (0.972–1.000)	100.00%	100.00%	100.00%	100.00%	100.00%
	Neural Network	0.940 (0.901–0.979)	83.3% (0.759–0.893)	61.91%	93.75%	80.00%	60.00%	97.56%
Test	Bagged AdaBoost	0.927 (0.834– 1.000)	87.5% (0.710–0.965)	71.43%	100.00%	83.33%	66.67%	100.00%
	Stochastic Gradient Boosting	0.865 (0.724–1.000)	81.3% (0.636–0.928)	57.14%	87.50%	79.17%	58.33%	95.00%
	Random Forest	0.906 (0.776–1.000)	87.5% (0.710–0.965)	69.23%	87.50%	87.50%	70.00%	95.45%
	Neural Network	0.927 (0.836– 1.000)	84.4% (0.672–0.947)	65.52%	100.00%	79.17%	61.54%	100.00%

PPV, Positive Predictive Value; NPV, Negative Predictive Value.

The conclusions yielded by analysis of *ceteris paribus* profiles and accumulated-local profiles are presented in **Supplementary Figures S2**–**S5**. *Ceteris paribus* profiles and accumulated-local profiles of each chosen feature displayed a comparatively consistent pattern in the vast majority of scenarios, intimating that they could precisely evaluate the behavioral patterns of the predicted value of the model as a function of a specified feature and conduce to the model’s facility for classification.

## 4 Discussion

In our research, we further investigated the potential utility of radiomics features to prognosticate the progression of patients with WHO grade II meningiomas within 5 years. The results intimated that the models based on radiomics features had promising value in predicting PFS.

WHO grade II meningiomas are also referred to as atypical meningiomas (2). In the 2007 WHO guidelines, brain invasion was one of the criteria for WHO grade II meningiomas, but merely as a staging criterion (5). However, in the 2016 WHO guidelines, brain invasion, due to its high correlation with the risk of recurrence and death, is separately listed as the diagnostic standard of WHO grade II meningioma, unchanged in the 2021 WHO guidelines (2, 41). Since the diagnostic criterion of WHO grade II meningioma was revised in the 2016 WHO guidelines, the proportion of WHO grade II meningioma has increased from approximately 5% to 15%–20% (5, 42). The sharp increase in the number of patients with WHO grade II meningiomas has led to greater heterogeneity. Previous studies on WHO grade II meningiomas may not be applicable, warranting further investigation. Although our study included some patients with meningiomas diagnosed before 2016, we reclassified all patients according to the 2021 WHO guidelines.

Previous studies demonstrated that 30%–60% of WHO grade II meningiomas progressed within 5 years after surgery (6, 8, 43). It is widely accepted that the extent of resection is the most relevant factor for postoperative progression (8, 44). Furthermore, age, postoperative radiotherapy, and tumor location have all been reported to be associated with postoperative progression of WHO grade II meningiomas (8–10). In our study, a significant correlation was found between age and PFS, consistent with most previous studies. Regarding gender, previous studies’ results vary (10, 45, 46). In our research, we found that male patients had a significantly shorter PFS time than female patients. In the majority of studies, GTR is an independent factor affecting postoperative PFS in WHO grade II meningiomas, corroborated again by our conclusion (6). There are relatively few studies that investigated peritumoral edema and postoperative PFS of WHO grade II meningioma. These studies revealed the trend that larger peritumoral edema is related to shorter postoperative PFS time (47, 48); our study exhibited a similar trend that patient with EI>2.3 had significant shorter PFS than patient with EI<2.3. Notably, in previous studies on peritumoral edema, a scale or approximate volume calculation was used to evaluate the volume of peritumoral edema, while our study delineated volumes of interest to accurately calculate the volume of peritumoral edema and tumor, and used the EI as the evaluation index, which is more accurate and scientific to some extent. Regarding tumor location, convexity and non-parasagittal meningiomas achieve higher postoperative PFS in our study, consistent with earlier research (45). Some studies suggest that skull base meningiomas are associated with shorter PFS time, which may be related to the difficulty of Simpson I resection of skull base meningiomas (46, 49), while some studies had found that the PFS of WHO grade II meningioma was unrelated to location (48). In our study, skull base meningiomas did not show a significant correlation with postoperative PFS of WHO grade II meningiomas.

The Wavelet transform decomposes three-dimensional data into various frequency components along three axes. In our previous work, the inclusion of Wavelet-based features has been proven to enhance the performance of prediction models (28). In this study, we found that most of the selected features were Wavelet-based features, further confirming the importance of Wavelet-based features for radiomic studies.

In our research results, the model based on radiomic features demonstrates good predictive ability for progression of WHO grade II meningioma within 5 years after surgery, exhibiting the unique advantages of radiomic analysis methods in predicting the prognosis of WHO grade II meningioma. At present, few studies exist on the relationship between radiomic features and postoperative progression-free survival of WHO grade II meningioma. Kalasauskas et al. enrolled 76 patients with WHO grade II meningioma and developed a model for identifying high risk of recurrence by combining radiologic semantic features with radiomic features (23). However, relatively few radiomic features were included, and machine learning algorithms were not exploited. Morin et al. investigated prognosis of meningioma by building predictive model based on clinical, radiologic, and radiomic features, which achieved the AUC of 0.78 when predicting overall survival (24). Omaditya et al. enrolled 43 WHO grade II meningioma patients and developed a radiomics model to predict Ki-67, which is related to PFS in meningioma patients. Recently, Park et al. conducted research to predict WHO grade II meningioma recurrence and identify patients who may benefit from adjuvant radiation therapy. Our study enrolled a relatively large sample size of patients with WHO grade II meningioma and fully exploit the advantages of radiomic features and artificial intelligence in predicting prognosis. The final model achieved a superior predictive performance. Based on the model, the PFS of WHO grade II meningioma can be better predicted. There is still controversy over whether radiotherapy is necessary after total resection of WHO grade II meningioma. In clinical practice, establishing a more accurate prognosis prediction model may help screen patients with high-risk of recurrence for radiotherapy and conduct closer follow-up examinations, which can help formulate personalized treatment plans for WHO grade II meningioma patients.

### 4.1 Limitations

A retrospective study design inevitably has inherent limitations. Foremost, insufficient complete data resulted in excluding a substantial number of WHO grade II meningioma patients from the cohort, which may undermine the performance of the predictive model. Second, although minimized as much as feasible, selection bias cannot be fully precluded. Third is the heterogeneity of imaging data; while a sequence of image preprocessing processes encompassing N4ITK and SUSAN algorithms was implemented to enhance data homogeneity and reproducibility of our study, given that the MRI data came from multiple disparate MRI scanning devices with different scanning procedures, these systematic errors cannot be wholly overcome. Fourth, this was a single-center study; a high-quality multicenter prospective study with a larger sample size assimilating more clinical features is justified to confirm our conclusions.

## 5 Conclusion

Clinical features including age, gender, tumor location, peritumoral edema, and extent of resection are substantially correlated with postoperative progression of WHO grade II meningiomas. Machine learning models evinced propitious performance when prognosticating PFS of WHO grade II meningiomas. Among them, the Bagged AdaBoost model and the Neural Network model demonstrated the optimal predictive ability. Our study posits that radiomic features from preoperative multi-parameter magnetic resonance images could assist in predicting prognosis of patients with WHO grade II meningiomas who underwent surgery, thereby bestowing benefit for patients in clinical practice.

## Data availability statement

The raw data supporting the conclusions of this article will be made available by the authors, without undue reservation.

## Ethics statement

The studies involving humans were approved by Second Affiliated Hospital of Zhejiang University School of Medicine. The studies were conducted in accordance with the local legislation and institutional requirements. Written informed consent for participation was not required from the participants or the participants’ legal guardians/next of kin in accordance with the national legislation and institutional requirements.

## Author contributions

Conceptualization, QZ; methodology, QZ, ZG, and ZT; software, ZG; validation, ZG and ZT; formal analysis, ZG and ZT; investigation, ZT and PX; resources, ZT; data curation, FD; writing—original draft preparation, ZG and ZT; writing—review and editing, QZ; visualization, FS; supervision, QZ and JZ; project administration, QZ; funding acquisition, QZ and CL. All authors contributed to the article and approved the submitted version.

## References

[1] Ostrom QT Patil N Cioffi G Waite K Kruchko C Barnholtz-Sloan JS. CBTRUS statistical report: primary brain and other central nervous system tumors diagnosed in the United States in 2013–2017. Neuro-oncology. (2020) 22:iv1–iv96. doi: 10.1093/neuonc/noaa200 33123732 PMC7596247

[2] Louis DN Perry A Wesseling P Brat DJ Cree IA Figarella-Branger D. The 2021 WHO classification of tumors of the central nervous system: a summary. Neuro-oncology. (2021) 23:1231–51. doi: 10.1093/neuonc/noab106 PMC8328013 34185076

[3] Bondy M Ligon BL. Epidemiology and etiology of intracranial meningiomas: a review. J Neurooncol. (1996) 29:197–205. doi: 10.1007/bf00165649 8858525

[4] Ayerbe J Lobato RD de la Cruz J Alday R Rivas JJ Gómez PA. Risk factors predicting recurrence in patients operated on for intracranial meningioma. A multivariate analysis. Acta Neurochir. (1999) 141:921–32. doi: 10.1007/s007010050398 10526073

[5] Louis DN Ohgaki H Wiestler OD Cavenee WK Burger PC Jouvet A. The 2007 WHO classification of tumours of the central nervous system. Acta Neuropathol. (2007) 114:97–109. doi: 10.1007/s00401-007-0243-4 17618441 PMC1929165

[6] Zhao L Zhao W Hou Y Wen C Wang J Wu P. An overview of managements in meningiomas. Front Oncol. (2020) 10:1523. doi: 10.3389/fonc.2020.01523 32974188 PMC7473392

[7] Goldbrunner R Minniti G Preusser M Jenkinson MD Sallabanda K Houdart E. EANO guidelines for the diagnosis and treatment of meningiomas. Lancet Oncol. (2016) 17:e383–91. doi: 10.1016/S1470-2045(16)30321-7 27599143

[8] Keric N Kalasauskas D Freyschlag CF Gempt J Misch M Poplawski A. Impact of postoperative radiotherapy on recurrence of primary intracranial atypical meningiomas. J Neurooncol. (2020) 146:347–55. doi: 10.1007/s11060-019-03382-x 31900826

[9] Shakir SI Souhami L Petrecca K Mansure JJ Singh K Panet-Raymond V. Prognostic factors for progression in atypical meningioma. J Neurosurg. (2018) 129:1240–8. doi: 10.3171/2017.6.Jns17120 29350599

[10] Masalha W Heiland DH Franco P Delev D Haaker JG Schnell O. Atypical meningioma: progression-free survival in 161 cases treated at our institution with surgery versus surgery and radiotherapy. J Neurooncol. (2018) 136:147–54. doi: 10.1007/s11060-017-2634-2 29081038

[11] Sahm F Schrimpf D Olar A Koelsche C Reuss D Bissel J. TERT promoter mutations and risk of recurrence in meningioma. J Natl Cancer Inst. (2016) 108(5). doi: 10.1093/jnci/djv377 PMC4849806 26668184

[12] Schmidt M Mock A Jungk C Sahm F Ull AT Warta R. Transcriptomic analysis of aggressive meningiomas identifies PTTG1 and LEPR as prognostic biomarkers independent of WHO grade. Oncotarget. (2016) 7:14551–68. doi: 10.18632/oncotarget.7396 PMC4924735 26894859

[13] Lambin P Rios-Velazquez E Leijenaar R Carvalho S van Stiphout RG Granton P. Radiomics: extracting more information from medical images using advanced feature analysis. Eur J Cancer (Oxford England: 1990). (2012) 48:441–6. doi: 10.1016/j.ejca.2011.11.036 PMC4533986 22257792

[14] Gillies RJ Kinahan PE Hricak H. Radiomics: images are more than pictures, they are data. Radiology. (2016) 278:563–77. doi: 10.1148/radiol.2015151169 PMC4734157 26579733

[15] Aerts HJ. The potential of radiomic-based phenotyping in precision medicine: A review. JAMA Oncol. (2016) 2:1636–42. doi: 10.1001/jamaoncol.2016.2631 27541161

[16] Laukamp KR Shakirin G Baeßler B Thiele F Zopfs D Große Hokamp N. Accuracy of radiomics-based feature analysis on multiparametric magnetic resonance images for noninvasive meningioma grading. World Neurosurgery. (2019) 132:e366–e90. doi: 10.1016/j.wneu.2019.08.148 31476455

[17] Zhu Y Man C Gong L Dong D Yu X Wang S. A deep learning radiomics model for preoperative grading in meningioma. Eur J Radiol. (2019) 116:128–34. doi: 10.1016/j.ejrad.2019.04.022 31153553

[18] Chen C Guo X Wang J Guo W Ma X Xu J. The diagnostic value of radiomics-based machine learning in predicting the grade of meningiomas using conventional magnetic resonance imaging: A preliminary study. Front Oncol. (2019) 9:1338. doi: 10.3389/fonc.2019.01338 31867272 PMC6908490

[19] Hale AT Stonko DP Wang L Strother MK Chambless LB. Machine learning analyses can differentiate meningioma grade by features on magnetic resonance imaging. Neurosurg Focus. (2018) 45:E4. doi: 10.3171/2018.8.FOCUS18191 30453458

[20] Hu J Zhao Y Li M Liu J Wang F Weng Q. Machine learning-based radiomics analysis in predicting the meningioma grade using multiparametric MRI. Eur J Radiol. (2020) 131:109251. doi: 10.1016/j.ejrad.2020.109251 32916409

[21] Yan P-F Yan L Hu T-T Xiao D-D Zhang Z Zhao H-Y. The potential value of preoperative MRI texture and shape analysis in grading meningiomas: A preliminary investigation. Trans Oncol. (2017) 10:570–7. doi: 10.1016/j.tranon.2017.04.006 PMC5487245 28654820

[22] Park YW Oh J You SC Han K Ahn SS Choi YS. Radiomics and machine learning may accurately predict the grade and histological subtype in meningiomas using conventional and diffusion tensor imaging. Eur Radiol. (2019) 29:4068–76. doi: 10.1007/s00330-018-5830-3 30443758

[23] Kalasauskas D Kronfeld A Renovanz M Kurz E Leukel P Krenzlin H. Identification of high-risk atypical meningiomas according to semantic and radiomic features. Cancers (Basel). (2020) 12:2942. doi: 10.3390/cancers12102942 33053798 PMC7599676

[24] Morin O Chen WC Nassiri F Susko M Magill ST Vasudevan HN. Integrated models incorporating radiologic and radiomic features predict meningioma grade, local failure, and overall survival. Neurooncol Adv. (2019) 1:vdz011–vdz. doi: 10.1093/noajnl/vdz011 PMC6777505 31608329

[25] Gennatas ED Wu A Braunstein SE Morin O Chen WC Magill ST. Preoperative and postoperative prediction of long-term meningioma outcomes. PloS One. (2018) 13:e0204161. doi: 10.1371/journal.pone.0204161 30235308 PMC6147484

[26] Li LM Zheng WJ Chen YZ Hu ZH Liao W Lin QC. Predictive factors of postoperative peritumoral brain edema after meningioma resection. Neurol India. (2021) 69:1682–7. doi: 10.4103/0028-3886.333500 34979669

[27] Adegbite AB Khan MI Paine KW Tan LK. The recurrence of intracranial meningiomas after surgical treatment. J Neurosurg. (1983) 58:51–6. doi: 10.3171/jns.1983.58.1.0051 6847909

[28] Guo Z Tian Z Shi F Xu P Zhang J Ling C. Radiomic features of the edema region may contribute to grading meningiomas with peritumoral edema. J Magn Reson Imaging. (2023) 58(1):301–10. doi: 10.1002/jmri.28494 36259547

[29] Tustison NJ Avants BB Cook PA Zheng Y Egan A Yushkevich PA. N4ITK: improved N3 bias correction. IEEE Trans Med imaging. (2010) 29:1310–20. doi: 10.1109/tmi.2010.2046908 PMC3071855 20378467

[30] Smith SM Brady JM. SUSAN—a new approach to low level image processing. Int J Comput Vision. (1997) 23:45–78.

[31] Pati S Singh A Rathore S Gastounioti A Bergman M Ngo P. The cancer imaging phenomics toolkit (CaPTk): technical overview. Brainlesion. (2020) 11993:380–94. doi: 10.1007/978-3-030-46643-5_38 PMC7402244 32754723

[32] Davatzikos C Rathore S Bakas S Pati S Bergman M Kalarot R. Cancer imaging phenomics toolkit: quantitative imaging analytics for precision diagnostics and predictive modeling of clinical outcome. J Med Imaging (Bellingham Wash). (2018) 5:011018. doi: 10.1117/1.Jmi.5.1.011018 PMC5764116 29340286

[33] Rohlfing T Zahr NM Sullivan EV Pfefferbaum A. The SRI24 multichannel atlas of normal adult human brain structure. Hum Brain mapping. (2010) 31:798–819. doi: 10.1002/hbm.20906 PMC2915788 20017133

[34] Perinetti G. StaTips Part IV: Selection, interpretation and reporting of the intraclass correlation coefficient. South Eur J Orthodontics Dentofacial Res. (2018) 5(1). doi: 10.5937/sejodr5-17434

[35] Chen Y Chen TW Wu CQ Lin Q Hu R Xie CL. Radiomics model of contrast-enhanced computed tomography for predicting the recurrence of acute pancreatitis. Eur Radiol. (2019) 29:4408–17. doi: 10.1007/s00330-018-5824-1 30413966

[36] Sunil KC Ramasree RJ. Dimensionality reduction in automated evaluation of descriptive answers through zero variance, near zero variance and non frequent words techniques - a comparison. In: 2015 IEEE 9th International Conference on Intelligent Systems and Control (ISCO). IEEE (2015).

[37] Box GEP Tidwell PW Tidwell PW. Taylor & Francis online: transformation of the independent variables - technometrics, Vol. 4. London: Taylor & Francis. (1962).

[38] Peng H Long F Ding C. Feature selection based on mutual information: criteria of max-dependency, max-relevance, and min-redundancy. United States: IEEE Transactions on Pattern Analysis & Machine Intelligence (2005). 10.1109/TPAMI.2005.159 16119262

[39] Ling CX Li C. Data mining for direct marketing: problems and solutions. Berlin, Heidelberg: AAAI Press (1998).

[40] Friedman J. Greedy function approximation: A gradient boosting machine. Ann Statist. (2001) 29(5):1189–232. doi: 10.1214/aos/1013203451

[41] Louis DN Perry A Reifenberger G von Deimling A Figarella-Branger D Cavenee WK. The 2016 world health organization classification of tumors of the central nervous system: a summary. Acta Neuropathol. (2016) 131:803–20. doi: 10.1007/s00401-016-1545-1 27157931

[42] Kshettry VR Ostrom QT Kruchko C Al-Mefty O Barnett GH Barnholtz-Sloan JS. Descriptive epidemiology of World Health Organization grades II and III intracranial meningiomas in the United States. Neuro Oncol. (2015) 17:1166–73. doi: 10.1093/neuonc/nov069 PMC4490879 26008603

[43] Maillo A Orfao A Espinosa AB Sayagués JM Merino M Sousa P. Early recurrences in histologically benign/grade I meningiomas are associated with large tumors and coexistence of monosomy 14 and del(1p36) in the ancestral tumor cell clone. Neuro Oncol. (2007) 9:438–46. doi: 10.1215/15228517-2007-026 PMC1994101 17704362

[44] Cao X Hao S Wu Z Wang L Jia G Zhang L. Treatment response and prognosis after recurrence of atypical meningiomas. World Neurosurg. (2015) 84(4):1014–9. doi: 10.1016/j.wneu.2015.05.032 26038336

[45] Ildan F Erman T Göçer AI Tuna M Bağdatoğlu H Cetinalp E. Predicting the probability of meningioma recurrence in the preoperative and early postoperative period: a multivariate analysis in the midterm follow-up. Skull Base. (2007) 17:157–71. doi: 10.1055/s-2007-970554 PMC1888737 17973029

[46] Lee JH Kim OL Seo YB Choi JH. Prognostic factors of atypical meningioma: overall survival rate and progression free survival rate. J Korean Neurosurg Soc. (2017) 60:661–6. doi: 10.3340/jkns.2017.0303.008 PMC5678060 29142625

[47] Simis A Pires de Aguiar PH Leite CC Santana PA Rosemberg S Teixeira MJ. Peritumoral brain edema in benign meningiomas: correlation with clinical, radiologic, and surgical factors and possible role on recurrence. Surg Neurology. (2008) 70:471–7. doi: 10.1016/j.surneu.2008.03.006 18586307

[48] Mantle RE Lach B Delgado MR Baeesa S Bélanger G. Predicting the probability of meningioma recurrence based on the quantity of peritumoral brain edema on computerized tomography scanning. J Neurosurg. (1999) 91:375–83. doi: 10.3171/jns.1999.91.3.0375 10470810

[49] Phonwijit L Khawprapa C Sitthinamsuwan B. Progression-free survival and factors associated with postoperative recurrence in 126 patients with atypical intracranial meningioma. World Neurosurgery. (2017) 107:698–705. doi: 10.1016/j.wneu.2017.08.057 28838877

