# Supplementary material for: Multi-parameter MRI radiomic features may contribute to predict progression-free survival in patients with WHO grade II meningiomas
Source: Front Oncol. 2024 Jun 28;14:1246730. doi: 10.3389/fonc.2024.1246730 (PMC11239420; doi:10.3389/fonc.2024.1246730)
Supplement: Supplementary file 1 [file DataSheet_1.docx]

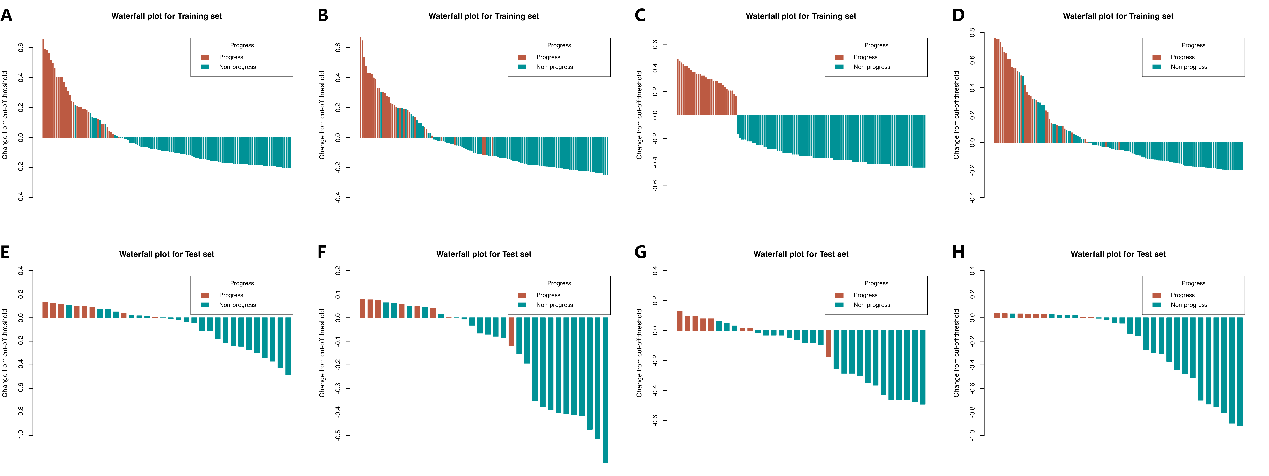


Supplementary Figure 1. Waterfall plots for models based on radiomic features. (A-D) Waterfall plots for (A) Bagged AdaBoost, (B) Stochastic Gradient Boosting, (C) Random Forest and (D) Neural Network models on training set. (E-H) Waterfall plots for (E) Bagged AdaBoost, (F) Stochastic Gradient Boosting, (G) Random Forest and (H) Neural Network models on test set. Threshold values were set as the zero point of y-axis. Cases were categorized as progress (upward bars) when the predicted values above the threshold, and vice versa. True classes of cases were given the red (progress) or green (non-progress) labels.


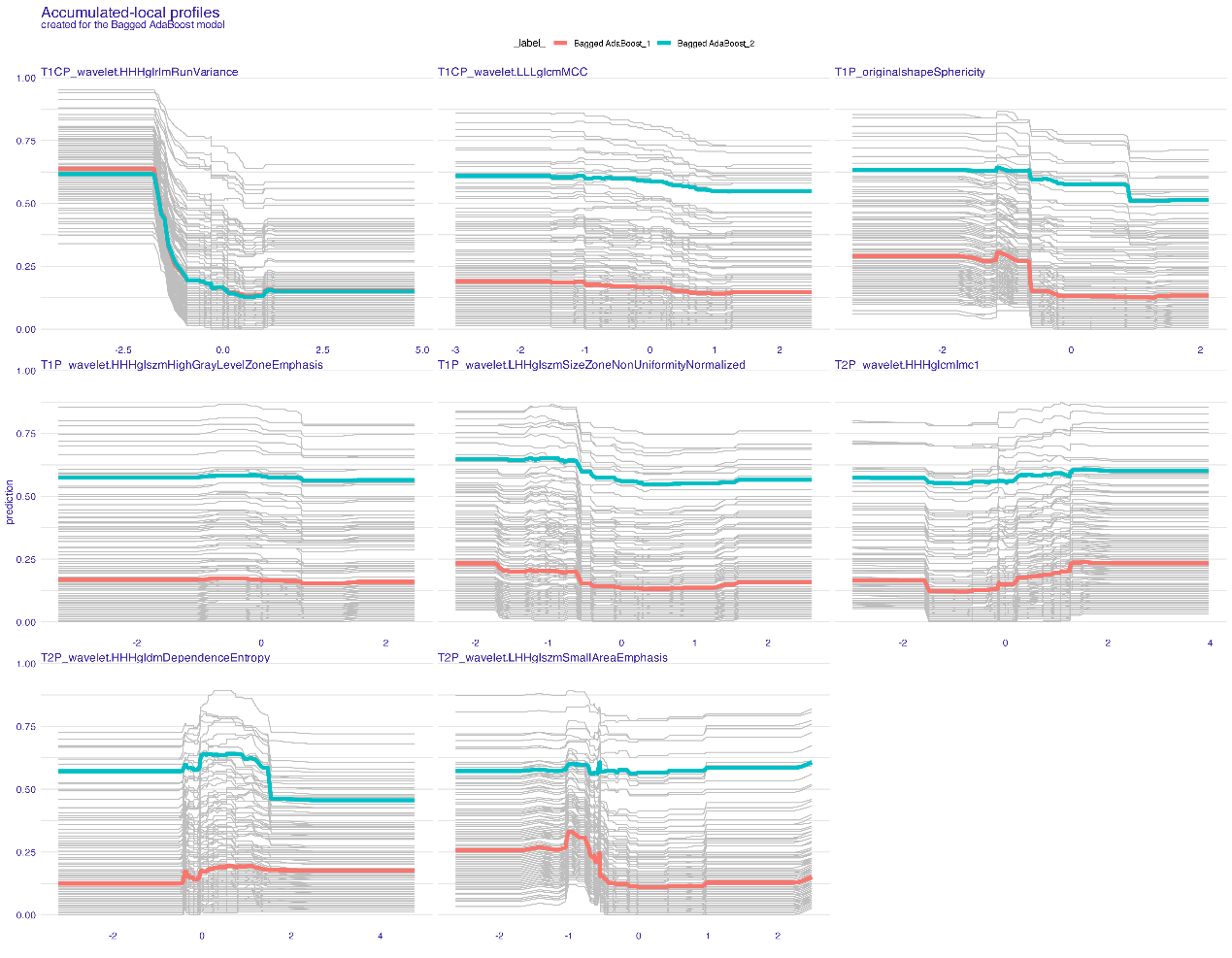


Supplementary Figure 2. Accumulated-local profiles for Bagged AdaBoost model based on radiomic features. The ceteris-paribus profiles for each case in the data set are represented by gray lines. Accumulated-local profiles in the colors red and green show two classes, respectively. Most of the gray lines are essentially parallel to one another. When compared to the gray lines, the red and green lines exhibited similar patterns and were relatively separated from each other.


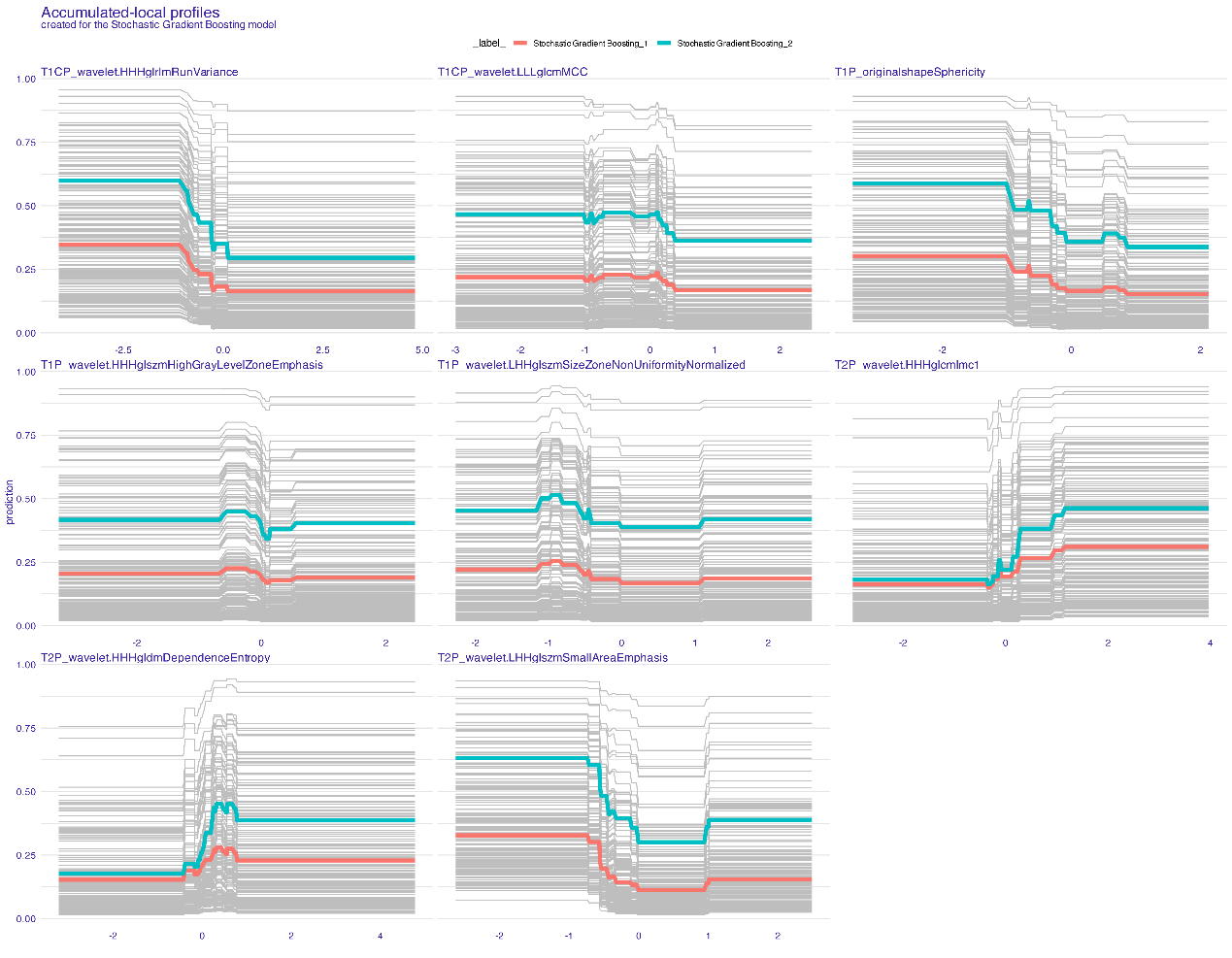


Supplementary Figure 3. Accumulated-local profiles for Stochastic Gradient Boosting model based on radiomic features. The ceteris-paribus profiles for each case in the data set are represented by gray lines. Accumulated-local profiles in the colors red and green show two classes, respectively. Most of the gray lines are essentially parallel to one another. When compared to the gray lines, the red and green lines exhibited similar patterns and were relatively separated from each other.


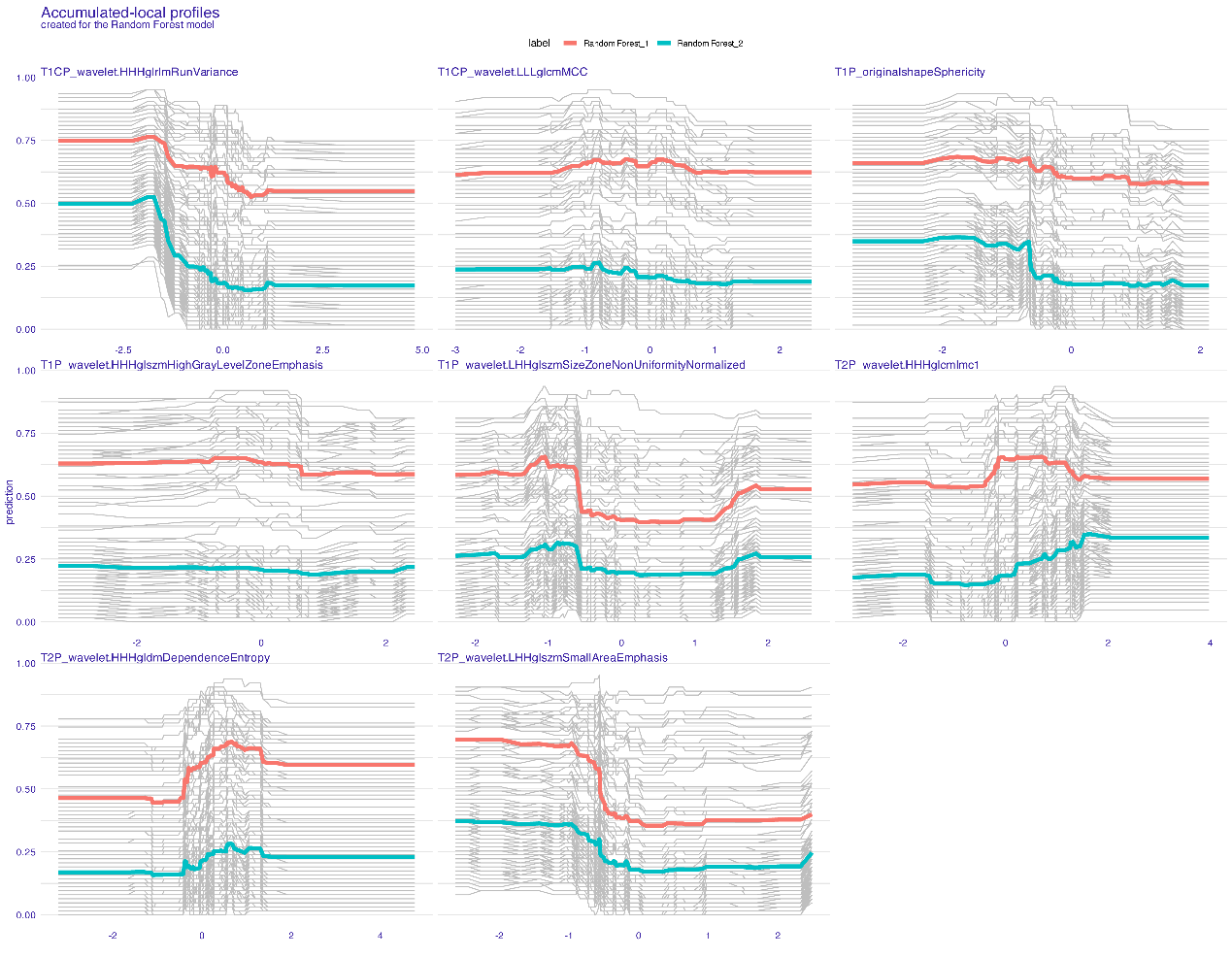


Supplementary Figure 4. Accumulated-local profiles for Random Forest model based on radiomic features. The ceteris-paribus profiles for each case in the data set are represented by gray lines. Accumulated-local profiles in the colors red and green show two classes, respectively. Most of the gray lines are essentially parallel to one another. When compared to the gray lines, the red and green lines exhibited similar patterns and were relatively separated from each other.


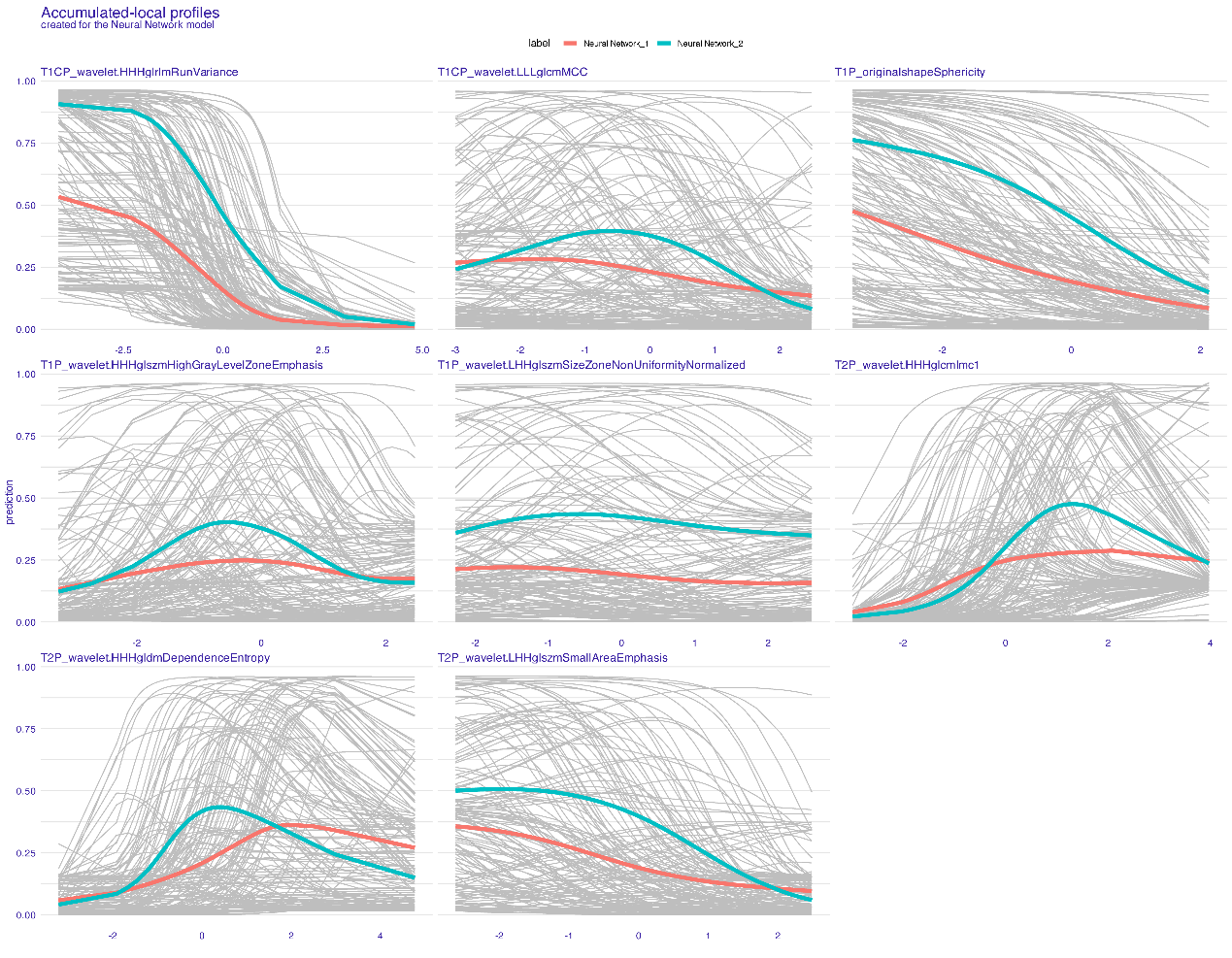


Supplementary Figure 5. Accumulated-local profiles for Neural Network model based on radiomic features. The ceteris-paribus profiles for each case in the data set are represented by gray lines. Accumulated-local profiles in the colors red and green show two classes, respectively. Most of the gray lines are essentially parallel to one another. When compared to the gray lines, the red and green lines exhibited similar patterns and were relatively separated from each other.
